# Pearls and Pitfalls in Cochlear Implantation

**DOI:** 10.1155/2011/438696

**Published:** 2011-12-29

**Authors:** Ingeborg Dhooge, Craig Buchman, Levent Sennaroglu, Emmanuel Mylanus, Françoise Denoyelle

**Affiliations:** ^1^Department of Otorhinolaryngology, Audiologic and Logopaedic Sciences, Ghent University, De Pintelaan 185, 9000 Ghent, Belgium; ^2^Department of Otolaryngology-Head and Neck Surgery, University of North Carolina at Chapel Hill, Chapel Hill, NC 27599, USA; ^3^Department of Otolaryngology, Hacettepe University Medical Faculty, Ankara, Turkey; ^4^Department of Otorhinolaryngology, Head and Neck Surgery, Radboud University Nijmegen Medical Centre, P.O. Box 9101, 6500 HB Nijmegen, The Netherlands; ^5^Service d'ORL et de Chirurgie Cervicofaciales Pédiatriques, Centre de Références des Malformations ORL Rares, Hôpital d'Enfants Armand-Trousseau, AP-HP, Université Paris VI-Pierre-et-Marie-Curie, 26, avenue du Dr-Arnold-Netter, 75012 Paris, France

Cochlear implantation (CI) has become the method of choice for the treatment of patients with bilateral deafness. With the development of technology and matured experience, candidacy is changing and becoming broader. Decision makers in charge of public health are faced with the decision for whom to include cochlear implants in the basic medical package. These decisions are based not only on the effectiveness of certain healthcare interventions but also on the costs that are involved. The first study systematically reviews the relevant studies on costs relating to cochlear implantation, analyzing the different methods used.

The second paper is an extensive literature review on the changing candidacy. Topics like cochlear implantation in very young children, cochlear implantation and hearing preservation, and cochlear implantation for unilateral deafness and tinnitus are discussed.

Since the introduction of universal newborn hearing screening, the vast majority of infants are screened for hearing loss within the first few days of life. Consequently, more patients are being detected with hearing loss early in life, and more paediatric patients are getting CIs in the course of their hearing rehabilitation. However, even with universal newborn hearing screening, still a number of deaf newborns are missed. As a result, some deaf infants receive late intervention and experience devastating language delays in which they spend a lifetime trying to catch up. The purpose of the third paper is to cross disciplines and to extend the understanding of the medical and audiological profession in seeing the language and literacy challenges deaf implanted children face whether they are early or late implanted.

Besides late implantation, other challenges, limitations, and potential risks remain in the treatment of deaf children with CI. Meningitis is still leading major causes of acquired severe sensorineural hearing loss. Labyrinthitis ossificans can lead to complete obliteration of the cochlea with limited possibility of cochlear implantation, consequently inducing poor auditory results. The 4th paper describes the audiological, anesthesiological and surgical key points of cochlear implantation after bacterial meningitis in very young infants.

With the increasing number of implant users worldwide, there is a growing need for assessing the overall survival of the various devices used and the safety and efficacy of reimplantation procedures. In paper 5 the authors investigate the effects of a perimodiolarly placed CI electrode on the cochlear microstructure. They also look at the possible damage to the modiolar wall at a microstructural level when pulling out an electrode. Based on their observations, they formulate some suggestions for a safe pull-out procedure.

The last paper highlights several areas where challenges remain for implant clinics, parents, and educators. While making clear the benefits of CI and its important role in extending opportunities for profoundly deaf children, challenges remain most notably in the areas of children's academic achievement and social development and participation with hearing peers. Ongoing attempts to address these challenges are essential if children with CI are to be fully supported to reach their potential personally, educationally, and socially.

*Ingeborg Dhooge* *Ingeborg Dhooge* *Craig Buchman* *Craig Buchman* *Levent Sennaroglu* *Levent Sennaroglu* *Emmanuel Mylanus* *Emmanuel Mylanus* *Françoise Denoyelle* *Françoise Denoyelle*

